# *Ehrlichia ewingii* Infection in White-Tailed Deer (*Odocoileus virginianus*)

**DOI:** 10.3201/eid0807.020018

**Published:** 2002-07

**Authors:** Michael J. Yabsley, Andrea S. Varela, Cynthia M. Tate, Vivien G. Dugan, David E. Stallknecht, Susan E. Little, William R. Davidson

**Affiliations:** * University of Georgia, Athens, Georgia, USA

**Keywords:** ehrlichiosis, zoonoses, southeastern United States, disease reservoirs, tick-borne diseases, deer, indirect immunofluorescence antibody test, PCR

## Abstract

Two closely related zoonotic ehrlichiae, *Ehrlichia chaffeensis* and *E. ewingii*, are transmitted by *Amblyomma americanum*, the lone star tick. Because white-tailed deer (*Odocoileus virginianus*) are critical hosts for all mobile stages of *A. americanum* and are important vertebrate reservoirs of *E. chaffeensis*, we investigated whether deer may be infected with *E. ewingii*, a cause of granulocytotropic ehrlichiosis in humans and dogs. To test for *E. ewingii* infection, we used polymerase chain reaction and inoculation of fawns with whole blood from wild deer. Of 110 deer tested from 20 locations in 8 U.S. states, 6 (5.5%) were positive for *E. ewingii*. In addition, natural *E. ewingii* infection was confirmed through infection of captive fawns. These findings expand the geographic distribution of *E. ewingii*, along with risk for human infection, to include areas of Kentucky, Georgia, and South Carolina. These data suggest that white-tailed deer may be an important reservoir for *E. ewingii*.

*Ehrlichia ewingii*, one of the causative agents of canine granulocytic ehrlichiosis, has been reported in dogs in several U.S. states, including Oklahoma, North Carolina, and Virginia (1–4). Human infections with *E. ewingii* have been reported from Missouri, Oklahoma, and Tennessee (5,6); the clinical disease, similar to that caused by other *Ehrlichia* spp., is characterized by fever, headache, and thrombocytopenia, with or without leukopenia (5–7). Experimentally, the lone star tick (*Amblyomma americanum*) has been shown to be a competent vector (8); however, natural infection of two other tick species, *Rhipicephalus sanguineus* and *Dermacentor variabilis*, has been reported in Oklahoma (2).

 The white-tailed deer (*Odocoileus virginianus*) is an important host for all three mobile stages of *A. americanum,* and deer and lone star ticks serve as the major reservoir and vector, respectively, for *E. chaffeensis* (9–11). Because *E. ewingii* is closely related to *E. chaffeensis* and shares the same vector, our goal was to determine if white-tailed deer are naturally infected with *E. ewingii*. In some human and canine infections with *E. ewingii*, cross-reactions with *E. chaffeensis* antigens have been reported (5,6); however, not all infections with *E. ewingii* result in positive serologic tests to *E. chaffeensis* antigen (2,6). Because *E. ewingii* has not been isolated in culture and because serologic test reagents are not readily available, we used several techniques to detect infections, including 1) testing serum samples for antibodies reactive with *E. chaffeensis* antigen, 2) testing leukocytes or whole blood by polymerase chain reaction (PCR) with primers specific for *E. ewingii* and *E. chaffeensis*, and 3) injecting captive white-tailed fawns with whole blood from deer collected in an *A. americanum–*endemic area.

## Methods

From September 1996 to July 2001, whole blood samples and serum from 110 deer from 20 sites (Table 1) in the southeastern United States were collected in vacutainer EDTA tubes (whole blood) and serum tubes (Becton, Dickinson and Company, Franklin Lakes, NJ). For PCR, two blood preparation protocols were followed. During the 1996–1997 collection period, leukocytes were separated from whole blood as described (9); during the 2000–2001 period, whole blood was extracted for PCR assays. Both leukocytes and whole blood samples were frozen at –20°C until PCR testing was done. Serum samples were held in vials at –20°C until serologic testing.

**Table 1:** Polymerase chain reaction (PCR) results for *Ehrlichia chaffeensis* and *E. ewingii* in 110 white-tailed deer, southeastern United States

Location^a^	County/state	*E. chaffeensis* PCR no. positive/ no. tested (%)	*E. ewingii* PCR no. positive/ no. tested (%)
White River NWR	Arkansas, AR	0/5	0/5
Felsenthal NWR	Ashley, AR	0/5	0/5
Pea Ridge NMP	Benton, AR	1/6 (17)	1/6 (17)
Shirey Bay WMA	Lawrence, AR	0/5	0/5
Cache River NWR	Monroe, AR	1/5 (20)	0/5
St. Vincent NWR	Franklin, FL	0/4	0/4
White Oak CC	Nassau, FL	0/5	0/5
Piedmont NWR	Jones, GA	0/5^b^	0/5^c^
St. Catherines Island	Liberty, GA	0/5	0/5
Blackbeard Island	McIntosh, GA	1/7 (14)	2/7 (29)
Harris Neck NWR	McIntosh, GA	0/5	0/5
Ballard WMA	Ballard, KY	0/5	0/5
Fort Knox	Hardin, KY	0/5	0/5
West Kentucky WMA	McCracken, KY	1/5 (20)	1/5 (20)
Tensas River NWR	Madison, LA	0/3	0/3
Dahomey NWR	Bolivar, MS	0/3	0/3
Cape Hatteras NS	Dare, NC	1/4 (25)	1/4 (25)
Mattamukseet NWR	Hyde, NC	1/5 (20)	0/5
Sea Pines	Beaufort, SC	0/18	1/18 (6)
Kiawah Island	Charleston, SC	0/5	0/5
Total		6/110 (5.5)	6/110 (5.5)

^a^NWR, National Wildlife Refuge; NMP, National Military Park; WMA, Wildlife Management Area; CC, Conservation Center; NS, National Seashore. ^b^At least 1 (20%) of 5 was positive based on transmission to fawn 81. ^c^At least 2 (40%) of 5 were positive based on transmission to both fawns 76 and 81.

Because *A.* *americanum* is the only experimentally proven vector for *E. ewingii*, locations with deer infested with *A. americanum* were selected for this study. Serum from each deer was tested for antibodies reactive to *E. chaffeensis* by the indirect immunofluorescent antibody (IFA) test as described (10), with the following modifications. Briefly, sera were screened at a dilution of 1:128 by using *E. chaffeensis* antigen slides obtained from Focus Technologies (formerly MRL Diagnostics, Cypress, CA). A 1:50 dilution of fluorescein isothiocyanate-labeled rabbit anti-deer immunoglobulin G (Kirkegaard & Perry Laboratories, Inc., Gaithersburg, MD) was used as conjugate.

 DNA from 200 µL whole blood or 20 µL leukocytes was extracted by using the GFX Genomic Blood DNA Purification Kit (Amersham Biosciences, Piscataway, NJ) and InstaGene Purification Matrix (Bio-Rad Laboratories, Hercules, CA), respectively, following the manufacturer’s protocol. Primary outside amplification consisted of 5 µL DNA from whole blood or 10 µL from leukocytes in a 25-µL reaction containing 10 mM Tris-Cl (pH 8.3), 50 mM KCl, 1.5 mM MgCl_2_, 0.2 mM each deoxynucleoside triphosphate (dNTP), and 2.5 units Taq DNA Polymerase (Promega Corp., Madison, WI), and 0.8 µM of primers ECC and ECB (11). For the nested PCR, 1 µL of primary product was used as template in a 25-µL reaction containing the same PCR components, except for the addition of *E. ewingii* specific primers, EE72-ewingii (5´-CAATTCCTAAATAGTCTCTGACTATT-3´) and HE3 (4), or *E. chaffeensis*-specific primers, HE1 and HE3 (11). Amplified products were separated in 2% agarose gels, stained with ethidium bromide, and visualized with UV light. Representative secondary PCR products for *E. ewingii* were purified with a Microcon spin filter (Amicon Inc., Beverley, MA), sequenced with an ABI 3100 automated sequencer (Applied Biosystems, Perkin Elmer Corp, Foster City, CA), and then compared with published *E. ewingii* sequences (GenBank accession nos. M73227 [3] and U96436 [1]).

 Two 4-month-old, laboratory-reared white-tailed fawns (76 and 81) were housed in a tick-free facility. Before inoculation both fawns were negative for antibodies reactive to *E. chaffeensis* and PCR-negative for both *E. chaffeensis* and *E. ewingii*. Whole blood for injection was obtained from five wild source deer (WTD 1–5) collected at Piedmont National Wildlife Refuge (NWR) in Jones County, Georgia, on July 24, 2001. A whole blood sample from each wild deer was also cultured in DH82 canine macrophage cells as described (12).

Fawns were anesthetized by intramuscular injection of tiletamine HCL and zolazepam HCL (4.4 mg/kg body weight; Fort Dodge Animal Health, Fort Dodge, IA) and xylaxine (2.2 mg/kg; Butler, Columbus, OH) and were reversed with intravenous injection of yohimbine (0.125 mg/kg; Lloyd Laboratories, Inc., Shenandoah, IA). Equal volumes of whole blood in EDTA from WTD1 and WTD2 were pooled, and a total of 8 mL was injected into fawn 76 in 2-mL aliquots by each of four routes (intravenous, intradermal, subcutaneous, and intraperitoneal). Fawn 81 was injected in the same way with a total of 8 mL of pooled blood from WTD3–5. Blood samples were collected from both fawns on 5, 9, 15, 20, 47, 68, and 110 days postinjection (DPI) for PCR, serologic tests, and blood smears. Blood was tested by PCR for *E. ewingii* and *E. chaffeensis* as described above and for the human granulocytic ehrlichiosis (HGE) agent (*Anaplasma phagocytophila*) by using primers GE9f and GA1UR, as described (13).

## Results

Ninety-seven (88.1%) of the 110 wild deer had antibodies reactive (≥1:128 titer) to *E. chaffeensis* by IFA testing. All locations examined contained seropositive deer (range 57%–100%). A 407-bp product characteristic of *E. ewingii* was generated in six (5.5%) deer by nested PCR, and six (5.5%) deer were also positive for *E. chaffeensis* (Table 1). Positive PCR results for *E. chaffeensis* and *E. ewingii* were obtained with both blood preparation processes. Only one deer (0.9%) was positive for both *E. ewingii* and *E. chaffeensis* by PCR.

 All five source deer (WTD1–5) were positive for antibodies to *E. chaffeensis,* but negative by PCR for *E. ewingii* and *E. chaffeensis* (Table 2). However, blood from deer WTD5 was culture positive for *E. chaffeensis*. Fawn 81 was at first positive for antibodies reactive to *E. chaffeensis* at 15 DPI, tested negative at 20 DPI, and was positive at 47, 68, and 110 DPI. Fawn 76 was seronegative on all days tested. Both fawns were PCR positive for *E. ewingii* at 47 DPI, and fawn 81 remained PCR positive at 68 DPI (Table 2). Whole blood samples from fawn 81 were PCR positive for *E. chaffeensis* at 15, 20, 47, 68, and 110 DPI. On thin blood smears taken at 47 DPI, morulae characteristic of *E. ewingii* were observed in approximately 2%-3% and <1% of neutrophils of fawns 81 and 76, respectively (Figure). Both deer remained PCR negative for the HGE agent.

**Table 2:** Summary serologic and polymerase chain reaction (PCR) data for fawns injected with pooled blood from infected source white-tailed deer^a^ (WTD1–5)

Fawns	IFA results	PCR results		
		*E. ewingii* (DPI) ^b^	*E. chaffeensis* (DPI)	HGE
Fawn 76 (received blood from WTD1–3)	—	+ (47)	—	—
Fawn 81 (received blood from WTD 4–5)	+	+ (47, 68)	+ (15, 20, 47, 68, 110)	—

^a^The five source deer (WTD 1–5) were positive by indirect immunofluorescent antibody (IFA) test (titer ≥128) and negative by PCR for *Ehrlichia ewingii, E. chaffeensis*, and the HGE agent (*Anaplasma phagocytophila*) ^b^DPI, days post inoculation; HGE, human granulocytotropic ehrlichiosis

**Figure:**
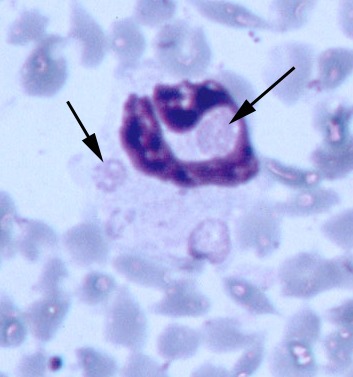
Multiple morulae consistent with *Ehrlichia ewingii* in a neutrophil from fawn 81 experimentally injected with pooled whole blood from two wild white-tailed deer (Giemsa stain, 100X).

 Sequences of three *E. ewingii* products (Dare County, North Carolina; Fawn 76; and Fawn 81) were identical to published gene sequences M73227 and U96436. The *E. ewingii* product from Benton County, Arkansas, differed from the others at base 225, which corresponds to GenBank accession number AY093439. The *E. ewingii* sequences were deposited in the GenBank database under accession numbers AY093439–AY093441 and AY497628.

## Discussion

Our data provide the first evidence that white-tailed deer are naturally infected with *E. ewingii*; this information extends the geographic distribution of *E. ewingii* to include areas of Kentucky, Georgia, and South Carolina. Before this report, the only reported vertebrate hosts for *E. ewingii* were humans and dogs. By combining data from PCR and injection studies, we showed that at least 8 (7.3%) of 110 deer were infected with *E. ewingii*, which is similar to prevalence rates previously reported for dogs. Infection with *E. ewingii* has been reported in 6.2%-15.8% of dogs from southeastern Virginia, Oklahoma, and southeastern North Carolina (2,4,14). Because of the limited sensitivity of PCR for detection of this organism, this percentage may represent a substantial underestimation of the actual prevalence of *E. ewingii* infection in white-tailed deer. Our data suggest that the distribution of *E. ewingii* and hence the risk for human and canine infection may be more widespread than previously reported and may correspond with the distribution of *A. americanum*.

 Although whole blood samples from all five deer (WTD1–5) collected at Piedmont NWR in Georgia were negative by PCR, *Ehrlichia* spp. infections developed in both inoculated fawns. Therefore, at least two of the Piedmont NWR deer were infected with *E. ewingii,* since *E. ewingii* infection was identified in both fawns. In addition, at least one Piedmont NWR deer was positive for *E. chaffeensis*, as fawn 81 became infected and WTD5 was culture positive. Because a much smaller volume of blood was used for PCR (20–200 µL) than for culture (5 mL) or injection of fawns (8 mL), low numbers of organisms may have been more readily detected by the other two methods. Consistent with results of previous studies (12,15), our data indicate that use of PCR alone as a screening tool may fail to detect acute infections of white-tailed deer with *Ehrlichia* spp*.*

Although fawn 76 was clearly infected with *E. ewingii* on the basis of PCR and detection of morulae, its results were never positive by serology. Serologic cross-reactions between *E. ewingii* and *E. chaffeensis* have been reported (5,6); however, not all *E. ewingii*-infected dogs or humans develop antibodies to *E. chaffeensis* antigens (2,6). Compared with previous experimental infections of white-tailed deer with *E. chaffeensis* (11,15), an extended period of time was required before *E. ewingii* was detected. Low numbers of *E. ewingii* in the original inoculum may explain the longer time required for PCR detection of *E. ewingii* in fawns 76 and 81. Because this experimental infection was a small pilot study, limited insight is provided into the course of *E. ewingii* infection in white-tailed deer. However, the detection of *E. ewingii* in fawn 81 over a 3-week period indicates that *E. ewingii* was capable of replicating in white-tailed deer.

White-tailed deer have been demonstrated as important reservoirs for *E. chaffeensis* (11,12,15). In this study, using PCR, culture, and inoculation of fawns, at least 7 (6.4%) of 110 deer were positive for *E. chaffeensis*. In previous studies in *A. americanum–*endemic areas, as many as 40%–100% of white-tailed deer have been shown to have antibodies reactive with *E. chaffeensis*, and up to 20% of deer are PCR positive (10,12). Five of the seven populations of white-tailed deer positive for *E. chaffeensis* were also positive for *E. ewingii*. This finding is not surprising, as these pathogens share the same vector. Although evidence of the HGE agent has been detected in white-tailed deer by both serologic testing and PCR (13,16), the relative importance of deer as reservoirs for the HGE agent has not been fully evaluated. Although our study demonstrates that white-tailed deer can harbor a third human ehrlichial pathogen, the importance of deer as a reservoir is not known.

Data from this study raise several important issues: 1) because of epidemiologic similarities between *E. chaffeensis* and *E. ewingii*, deer could be an important reservoir for *E. ewingii*; 2) because of potential serologic cross-reactivity, *E. chaffeensis* seroreactors in the current and prior surveys of white-tailed deer (10,17) could actually represent *E. chaffeensis*, *E. ewingii,* or mixed infections; and 3) because at least four *Ehrlichia* species infect white-tailed deer (*E. chaffeensis*, *E. ewingii*, *A. phagocytophila*, and an undescribed *Ehrlichia* sp.) (9,12,13,16), an array of diagnostic assays should be used for detecting *Ehrlichia* spp. infections. Therefore, further studies are needed to examine the reservoir potential of white-tailed deer for *E. ewingii* infection.
